# Stigma and Discrimination During COVID-19 Pandemic

**DOI:** 10.3389/fpubh.2020.577018

**Published:** 2021-01-12

**Authors:** Divya Bhanot, Tushar Singh, Sunil K. Verma, Shivantika Sharad

**Affiliations:** ^1^Department of Applied Psychology, Ramanujan College, University of Delhi, New Delhi, India; ^2^Department of Psychology, Banaras Hindu University, Varanasi, India; ^3^Department of Applied Psychology, Vivekananda College, University of Delhi, New Delhi, India

**Keywords:** COVID 19, stigma, stigmatization, discriminatory behaviors, victimization, social identity

## Abstract

The COVID-19 pandemic has been instrumental in creating a dramatic shift from people's need to live in mutual association toward a desire to stigmatize distinctive others. Pandemic seems to be causing othering. Stated simply, stigmatization is a social process set to exclude those who are perceived to be a potential source of disease and may pose threat to the effective social living in the society. Based on the secondary evidence collected from news published online or in print, the present article delves into stigma associated with the COVID-19 pandemic among different social groups in the Indian society and the mounting cases of prejudice based on race, class, and religion. It also presents insights into the varied manifestations, and the deleterious consequences of COVID-19 inspired othering brought to its potential targets in India.

## Introduction

Humanity today is facing one of the biggest challenges of the century. The novel coronavirus is spreading rapidly to the extent of being declared as a pandemic across the world. The spread of the COVID-19 pandemic has raised concerns of everyone across the globe. People are in dismay for what is happening with them and at the same time are disturbed to see the conditions of others, particularly the marginalized. There is a sudden shift in people's daily routines. Apart from the fears, anxiety, and sadness, people's sense of irritability has started piling up. Amid such a deranged spread of COVID-19, one of the important concerns that is even more deleterious than all the above highlighted negative impacts and needs to be urgently attended to is stigmatization associated with the pandemic.

People have been witnessed to undergo a dramatic shift from their willingness to live in mutual association to an urge to practice stigmatization (1) of individuals, groups, and nations who are comprehended as potential sources of virus contagion to others. In other words, the pandemic seems to be causing othering (2), manifesting at the global as well as at the local context leading to a tremendous loss of social capital. The stigmatizing behaviors in the present context are being guided by the famous adage “better safe than sorry” (3) that explain that how the fear of something unknown and uncertain (4) accounts for the negative attitudinal reactions directed toward the people who are infected or are suspected and the ones considered responsible for the spread of the virus.

The present article takes a look at the increasing cases of “othering” that are characterizing the societal response at large. The focus will be on different social groups that are the targets of prejudice and discrimination so rampant during the COVID-19 crisis in India. It includes prejudice based on religion, occupation, race, and economic class.

## The Psychology of Stigma

The term *stigma* was first introduced by Goffman (5) to refer to visible characteristic features (such as cut of burnt) of the individuals that make the society devalue and consider them unfit for their inclusion in the mainstream society. Subsequent scholars have attempted to define the term from their unique perspectives (6) explaining the term with respect to relationship between mark and discrediting dispositions (7), a sociocultural-driven phenomenon (8), interwined in the nexus of power dynamics (9), which function to reinforce the preexisting power differentials (10–12).

The stigmatization phenomenon has been the intriguing areas of exploration pertaining to the specific context in which it unfolds. The evolutionary approach to stigmatization provides a convincing answer to the origin of stigmatization (8). Stigmatization is practiced as an adaptation (13) following a principle of discriminate sociality (14–16) in the perception of danger, threat, or challenges to one's social living, and attempts are made henceforth to safeguard oneself from various such foreseen or unforeseen impediments such as getting prone to infectious diseases, being advocated to the values contrary to their own, and having an intimidating out-group, etc. (8, 17, 18). The stigma of COVID-19, in the present context, could be comprehended as a social process that sets to exclude those who are perceived to be a potential source of disease and may pose threat to the effective social living in the society (13, 19).

Several theoretical approaches provide explanations to the phenomenon of stigmatization and the way it folds. In the following sections, we will try to explain the origin of stigmatization, theoretical approaches, highlighting the unfolding of the phenomenon, the purpose it serves for the stigmatized, and the effects the stigmatized reap out of their experiences of negative attitudinal reactions of the society toward them.

One of the earliest theories, the social interactionist theory of stigma (5), talks about the negative self-conceptualizations held by the stigmatized when they comprehend a discrepancy, during social interactions, between what the society expects them to be and what they truly are. As a result, the stigmatized experience shame for not being able to meet the expectations of the society and experience anxiety and fear of being rejected by the society.

The labeling theory by Becker (20) explains that people attach labels to others in order to ease their understanding of their social world around. The theory explains stigmatization as a phenomenon unfolding against those who are labeled as deviant based on their specific attributes or behaviors perceived as contrary to the acceptable standards in the particular sociocultural framework. As a result, stereotypes are attached to the deviant labels (5, 9, 21), and the targets become the recipients of negative psychosocial and emotional reactions of the society, hence stigmatized (22). The chances of stigmatization are direct functions of power and resources of the targets, level of tolerance for the deviance by the society, social distance between the two, and visibility of the deviance (23).

Another explanation for stigmatization comes from social identity theory (24, 25), which draws it from the self-categorization theory (Turner, 1979). According to this theory, self-concept of individuals draws heavily from their belongingness to social groups (25), which gives rise to intergroup comparison (26). Emphasizing upon the superiority of one's own group, a phenomenon called ethnocentrism (27), people set to positively evaluate and favor the members of their own group (in-group) and engage in derogatory attitudinal reactions (stigmatization) against the out-group for it reaps them benefits of elated sense of self-esteem.

As against the previous theories that talk about the explanations for the unfolding of stigmatization in a particular sociocultural context, the model of stigma-induced identity threat (28) highlights the reactions of the stigmatized on being exposed to the derogatory treatments of the society. In addition to experiencing stress, the reactions of the stigmatized are influenced by the way they appraise or evaluate the stigmatizing situations based on their collective representations (awareness about one's stigmatized status in the society, the dominant stereotypes associated, and the recognition of being discriminated against) (29), immediate situational cues (the characteristics of the presenting situation that could be perceived in terms of the amount of threat it brings to the social identity of the stigmatized) (30), and individual characteristics (the personal characteristics of the stigmatized that catalyze the influence of the stressful situations on the stigmatized, like the extent to which they identify themselves with their stigmatized group–(31)). Identity threat results when the situation is appraised by the stigmatized as harmful and exceeds the coping resources available with them to overcome it, resulting in several voluntary and involuntary reactions.

The process of stigmatization has several benefits for the stigmatizers (32) that serve to explain why people stigmatize others. Stigmatization not only helps perceivers to form a holistic and a simplified understanding of the targets (33–36), but also allows them to go beyond the available information about the targets and make judgments about their personality and behaviors (37). Stigmatizers strive to cultivate their biological and reproductive fitness through stigmatizing the diseased (19), dominating and exploiting others (11, 12), for example, which aids a successful transfer of genes to the offspring (38, 39).

Stigmatization also helps stigmatizers in maintaining inequality through power differentials (19), preserving important resources for themselves (8, 40, 41), such as wealth, power, and a reputed status (19), exploiting the stigmatized to serve their purpose (19, 42, 43) and emphasizing control over them by practicing derogatory behaviors against them (44). These practices serve to boost the self-esteem and well-being of the stigmatizers, as well as serve to reduce their existential anxiety [Terror management theory by (45)].

Several studies in the past have studied the negative attitudinal reactions of the society against the stigmatized in relation to a number of physical and psychological health problems, such as AIDS (46), mental illnesses (16, 47, 48), facial disfigurement (49), cancer, leprosy, and physical disfigurement (50), and in relation to various sociological factors, such as homelessness (51), sexual orientation (52), social class (53), caste (54), etc., where the stigmatized become the passive recipients of negative emotional reactions from the powerful others (55).

Prejudices and discriminatory reactions against the stigmatized have also been the area of concern in the context of epidemics such as severe acute respiratory syndrome (SARS) (56) and H5N1 (57). Fear of contracting has been understood as one of the major precursors for the people to indulge in stigmatizing the infected (58) and the suspected because of their close-knit association with the spread of the disease [(56), p. 359]. Hatred is witnessed to be a common reaction of the society against the stigmatized during epidemics, particularly during modern times (59).

Although the stigma associated with pandemic has been a well-established phenomenon [(56), p. 359], due to its contextual nature (8, 13, 29, 60), the way it unfolds might vary depending on the context it finds its existence in.

The present ongoing situation of COVID-19 pandemic and its impact not only on the physical and psychological health but also on the way people are interacting with others are compelling enough to initiate analytical examination of stigma and discrimination related with COVID-19. This seems essential for the effective control of the disease, and the negative consequences of stigma aligned with being infected with coronavirus are extremely pernicious, the same way those were evident during SARS [(61), p. 729] and H5N1 outbreak (57). The psychological burden of such treatments strongly influences people's willingness to seek treatment or even let others know about (62). This not only impedes the process of effective management and minimization of the spread of the disease but also brings debilitating consequences for the overall well-being of the survivors and their relatives [(63), p. 108].

At this backdrop, using an analytical perspective, the present article delves into examining the way COVID-19–related stigmatization has unfolded itself against the specific sections of the Indian society and to gain a holistic understanding of the experiences of stigmatization as experienced by the people after the outbreak COVID-19 pandemic. An understanding of these would help us understand the way in which a crisis situation may lead to the segmented organization of the society in terms of strengthening of already existing categorizations, as well as emergence of new categorization. In addition, such an understanding is expected to supplement the government about the potential impediments that stigma is assumed to be creating in withholding the people's tendency of cause a delay in getting themselves tested or share their medical condition of being infected with coronavirus because of the fear of being stigmatized.

## Methods and Procedure

The present review is based on a careful review of literature on stigma during a pandemic and/or medical emergency and on the thematic analysis of news reports published online and/or in print editions since the outbreak of this pandemic in India. While the literature served as a vantage point to evaluate the social reactions of the current pandemic, the newspaper reports were treated as the sources of data related to the experience of stigma during COVID-19 pandemic. The news reports presenting negative reactions and/or experiences and the stigmatized treatments directed against people during the COVID-19 pandemic were thematically analyzed, and anecdotes were extracted to describe the stigma related to the dejection, derogation, devaluation, exclusion, discrimination, etc., associated with COVID-19.

## Stigma and Discrimination in India: Emerged Themes

From the careful analysis of the content extracted through the newspaper reports, several themes emerged indicating the stigmatic expressions and behaviors during the pandemic. The following section discusses these emergent themes in the light of the available literature explaining stigmatization.

## Stigmatization of the Suspected and Actually Infected Individuals

Since the outbreak of the pandemic in India, there existed a negative perception toward those infected with the disease. The COVID-19 patients are accused of being ignorant and negligent, thereby being held responsible for having contracted the virus (64). The COVID-19 patients were being stereotyped as the active spreaders of coronavirus and were being treated as the passive acquirers of the disease. Such a stereotype led the society to adopt several negative treatments (ranging from social media posts against them, stopping their entry into the residential areas, and spreading rumors against them on the basis of their religion, class, and caste) directed against them. Being an atypical condition, the devaluation associated with the mark of COVID-19 is indelible (19). Probably, that is why the consequences attached to its stigmatization are so devastating that even the formerly diagnosed continue to be stigmatized (65–67), and even after defeating the virus, they have not been able to free themselves from being shunned by society. They are reported being treated as untouchables, receiving the humiliating taunts, and fingers pointed against them and their family; their lane of residence has been named as “corona wali gali” (corona street), and the associated burden is strong enough that it has even compelled them to sell their own house (68). The fear among the people is so intense that it has led them to blame the scapegoats—especially the poor, laborers, daily wagers, and the migrants (69). Reports indicate that the people working in Delhi (India) and residing in Haryana (India) were negatively labeled as “corona carriers” by the Home Minister of Haryana, devaluing the former for the possible spread of the contagion (70). Even the doctors were not spared from being titled as the “carriers” of coronavirus (71). Therefore, not only the infected but even the suspected (due to the high risk of being infected) become the potential recipients of stigmatization (72).

There exists sufficient literature that highlights stigmatizing reactions of society against the infected during pandemics such as SARS outbreak [(56), p. 359] and shows that communicable negative health conditions bear stigma (73). The stigma toward the infected or feared to be infected with COVID-19 could be explained by the terror management theory (45). Because of the lack of any medication or vaccine available for COVID-19 (74), a lot of terror has been evident among the people at large (75). This heightened existential anxiety among the people acknowledging the possibilities of their mortality due to contagion with coronavirus (76) seems instrumental in triggering set of defenses in the form of negative attitudinal reactions against those who threaten people's belief in their immortality (77, 78) i.e., the COVID-19 infected, their associates, and the suspects of it.

The evolutionary perspective (8) of stigmatization also sets to explain such negative treatments against the COVID-19 infected. According to this perspective, the stigma serves as the means to avoid and make distance from the coronavirus contagious individuals or groups (1) to safeguard themselves from catching the infection (6, p. 58). Such attitudinal reactions could also be understood in the light of labeling theory (20). Because the infected are labeled as different based on their unacceptable medical condition, they reap stigmatization from the society.

The notion of double stigmatization was also seen in some cases. A deaf-mute, for example, was deserted by his own family after they suspected him to be coronavirus positive that was later confirmed as not bearing the virus (79). Similarly, a coronavirus-negative deceased elderly was abandoned by the family suspecting the former as highly vulnerable to catch the virus (80).

## The Stigma Based On Race: The Case of Northeast Indians

The easternmost part of India, comprising eight states—Assam, Sikkim, Arunachal Pradesh, Manipur, Nagaland, Meghalaya, Mizoram, and Tripura—is known as Northeast India. The residents from the Northeast India have been the targets of racism from the mainlanders in India for a long time as they have typical mongoloid features, which are similar to the features of Chinese individuals (81). The people of Northeast India have mostly borne the brunt of racism and discrimination and have been often regarded as foreigners in their own country. The racism toward Northeast Indians have heightened during the COVID-19 pandemic, and many cases were reported (81) where Northeasterners were called “corona” spat at, socially avoided, asked to vacate their houses by their landlords, beaten, suspended from employment, or had difficulty in accessing health care (82, 83). These racial attacks and discrimination have also been evident in the prestigious educational institutions of the nation including Kirorimal College, affiliated to University of Delhi, Tata Institute of Social Sciences, Mumbai, as well as National Council for Educational Research and Training, Delhi (84).

Literature reports racism to be an important factor leading to an undue spread of disease in the minority community (85) even leading to their deaths (86). However, the experience of stigma by Northeastern individuals in India has mostly been due to their association and facial similarities with Chinese individuals who are also stigmatized by many to be the reason for the origin of this pandemic.

## The Stigma Based on Profession: State of Health Care Professionals and Police Personnel

During this COVID-19 crisis worldwide, every country is trying to the best of its abilities and resources, to curb the spread of the pandemic. Individuals, groups, and communities are coming together and are presenting ultimate examples of prosocial behavior by helping those in need. Among them, there are doctors, nurses, other health care workers, police officers, and municipal workers who are risking their lives to serve their nation. India is no exception to this.

However, the doctors who are making arduous efforts to save the lives of the patients (87, 88) and the police officials who are working day and night, away from their families [(89–91)], are being ill-treated by the society (92, 93). They are fearful and are experiencing frustration arising out of their hard struggle and above all are facing social stigma (87, 94, 95) that does not even end with their death (96).

People at the forefront of the war against the pandemic (also called as coronavirus warriors) are becoming ostracized by their neighbors, landlords, taxi drivers, and even their own family members. Having left with no other option, doctors and nurses have had to sleep in the staff rooms and even in the washrooms of hospitals. Taxi drivers have also refused to serve them (97). The nurses have become homeless because of being shunned, attacked, and accused by their fearful landlords (87, 97) and have faced abusive and vulgar comments (98). Several cases of harassment (80), assaults, and false accusations of spreading the virus (99) have also surfaced against them. This has left them experiencing dismay (100), humiliation, and hurt, causing them to leave their homes (71).

Such ongoing stigmatized treatments directed against doctors, other health care workers, police officers, and municipal workers present classic representation of stigma by association (5, 101). Social stigma in this context becomes a function of disadvantageous alliances wherein even people who were not initially a part of the stigmatized group (doctors, nurses, and police) become the targets of stigma [because they are exposed the maximum to COVID infected patients; (102)].

## The Stigma Over the Death

The social stigma of COVID-19 has not even shown mercy to the dead bodies of the patients. There have been violent disruptions or prohibitions of funeral ceremonies (80) and burials (103, 104) of COVID-related deaths. Fearful officials of Nigambodh Crematorium in Delhi refused to perform the last rites of the infected dead bodies because of the sheer lack of knowledge about how the virus spreads (105). People in Chennai opposed the cremation of a doctor and assaulted the medical staff on duty (96, 106, 107) for the same reason. Residents of village Verka, Amritsar, had denied the cremation of Padam Shri Awardee and Hazoori Ragi, Mr. Nirmal Singh (108). Similarly, people in West Bengal protested against and condemned cremations of COVID-19 deaths at regular cremation places (109).

People's stigmatizing reactions for the dead family member indicate the strong and deep-seated embeddedness of irrational fear and threat that the virus has brought with itself. Several families in India denied claiming the dead body of their own kin members (110). Some have refused to do their last rites (111). Not even the ashes were collected by them, fearing the contagion; families forget about having their last glimpse before cremation (112).

Under such disturbing circumstances, many non-governmental organizations have taken the initiative of performing the last rites of the abandoned deceased. Abdul Bhai's Ekta Trust, for example, was accorded with the responsibility by the Surat Municipal Corporation, for the cremation and burial of COVID-19–infected bodies as per their respective religion (113).

## The Stigma Based On Religious Identity: MARKAZ Congregation

During March 13–15, 2020 a religious meeting (congregation) was held constituting of members of Tablighi Jamaat, an Islamic missionary and reformist organization (majorly of *Sunnis-*an Islamic subgroup), from all over the world at the Nizamuddin Markaz (Center) in Delhi. Later it was found that majority of these members were coronavirus-positive, and before they could be tested and contained, they returned back to their respective places all over India. The fear of the spread of the virus among general public was at the peak during that time, and the entire Muslim community at that time was stigmatized as the spreaders of the virus. The stigmatization of the whole Muslim community has been at the forefront of Indian public's reaction to COVID-19. Some political leaders were witnessed calling the Jamaat event as “corona terrorism” (114), and the congregation attendees as the “enemies of humanity” (115). Such reactions fueled the feelings of hatred and misplaced undue blame for the spread of the virus to this community.

In accordance with the dual model of impression formation (116) and the suppression justification model (117), holding a handful of Muslim Jamaatis' responsible for the spread of COVID-19 infections at augmented levels among the Indians could be contemplated as sufficient condition for cultivating the feeling of hatred and disgust for the whole Mohammedan community (114).

In accordance with the Social Identity theory (25), and Sumner's (27) conceptualization of ethnocentrism, the strong prejudices (118) exhibited against the social identity (119) of the Muslim community could be understood as a motivational act (120). Scheff (23) explains the level of tolerance for the target as a determining factor of the strength of the stigmatizing reactions directed against them. There has been a long history of Hindu-Muslim religious prejudice and discrimination reflecting less intergroup tolerance. The exaggerated negative reactions of society against the Muslim community could also be attributed to the role of media (121, 122). Sensationalized and inaccurate reporting, like showing doctored videos of Jamaat members spitting on others (114), has contributed to public hysteria and widespread negative perception of the Muslim community. The consequence was the surge in hostility, segregation, and violence projected toward the whole Muslim community (123) and twitter hashtags saying “corona jihad” (114).

These acts have functioned to validate that Muslims' subvert position is well-deserved (54, 101) and have contributed to the entrenchment of the already existing gaps that exist between the religious groups in the society (10, 101, 124, 125).

## Stigma Against the Migrant Workers

A significant chunk of the Indian population migrates from their villages to different states and cities in search of employment and work largely in the unorganized/informal sector. When the nationwide lockdown was suddenly announced as a quick response measure to curb the pandemic, the country was neither prepared nor had foreseen the consequences the lockdown would have for the migrant workers, daily wagers, laborers, house helps, street vendors, barbers, plumbers, mechanics, and many more. The lockdown was perhaps the first step toward othering. It had an inbuilt bias toward the privileged when it was presumed that people could stay locked up in their homes and survive, without considering the fact that how would migrant workers and daily wagers survive even for a day without work with their hand-to-mouth existence. And within a week of lockdown, India witnessed and continued to witness over months one of the biggest humanitarian crises—the mass migration of millions of workers propelled by their socioeconomic hardships. The poor migrant workers were left with no choice, but to leave for their hometowns. The central and state governments had not envisioned this mass exodus. However, with the public transport system being shut, they were compelled to use other modes such as bicycle and even foot, for covering distances of over thousands of miles. There were many videos and photographs circulating on news media, highlighting the suffering of the poor—exhausted men, women, and children, walking empty stomach, carrying their belongings, with the sun glaringly over their heads (126, 127). It is humiliating to become a kind of refugee in one's own country and have negligible social security. As pointed out by Gupta (128), white-collar workers and students who returned home after lockdown from overseas as well as other Indian states were not labeled as “migrants”; the label was reserved to refer to people belonging to the lower socioeconomic strata whom Gupta (128) calls “collarless workers.” The term “migrant” strategically paints a dehumanizing picture of these workers in mass media. Scheff (23) asserts that the chances of labeling specific kinds of people as deviant more than the others are the function of social distance between them and the society. This aptly explains the differential treatment poor received as compared to the white collar dominant others.

The stigma of being poor was highlighted when the government made arrangements for bringing back Indian students, tourists, and others who were stranded in foreign countries, but paid little attention to the plight of these workers. Other incidents were when the migrant workers were sprayed down with disinfectant by health/civic departments on two occasions (129, 130). The act was not only unreasonable but also highly undignified, highlighting stigmatization to be the function of the social status of the people (131). It objectified the poor workers as contaminated with the virus. It also mirrored the racist treatment received by Latinos on the American border, who too were dehumanized in the exact same way a few decades earlier. The predicament of the migrant workers strongly suggests how fear-ridden powerful systems victimize and blame the helpless marginalized groups.

## The Consequence of Stigma Due to COVID-19

The model of stigma-induced identity threat talks about the negative consequences stigmatization brings for the overall well-being of the people when they appraise the stigmatizing situations and identity threatening (28). The patients of COVID-19 are stigmatized and hence are bearing the consequences that are far more pernicious than the condition in its own self (1, 132). Social rejection has created a barricade between them and society (122) with repercussions for their physical, psychological health, and well-being (64). The patients are fearful of being shamed and stigmatized by society, extreme enough to exhibit the symptoms of hysteria (64). Some have also equated their distress to posttraumatic stress disorder (133).

The director of All India Institute of Medical Sciences, Delhi, warned about the perceived dread of being stigmatized among the people leading them to refrain from getting tested (134, 135). The social ostracism is responsible for people not seeking treatment or reporting symptoms and thus impeding an early detection of the virus and its effective control (62, 102, 122). The conditions are not even favorable for those who have tested negative for coronavirus. An individual in Madurai (136) and another one in Himachal Pradesh (**?** ) committed suicide on facing social boycott even after being tested negative for coronavirus.

A recent survey (137) revealed that 61% of people in India are suffering from mental health concerns, with the percentage of women outweighing that of men. The deteriorated psychological health was mainly attributed to the lockdown and the associated difficulties (137). Among others, one of the significant concerns raised by the National Commission for Women is the rising quantum of domestic violence cases in India amid lockdown (71, 138). While lockdown and social distancing enforced by the government in the prevailing pandemic have contributed to an extent in curbing the spread of the virus, it has also contributed in the people experiencing depression (139, 140), anxiety, terror, panic, heart disease arising out of loneliness (140), and committing suicides (141). People are apprehensive about the possibilities of unknowingly carrying the coronavirus (142–144). All these ill effects of the pandemic when associated with the rising stigmatization and discrimination are expected to have far-reaching consequences for the Indian society.

Other stressful concerns of people include witnessing the difficulties of the underprivileged and facing the economic crisis, increased frustration with other people, disordered regime, unpredictable future and the virus itself (145), maintaining physical distance, curtailed travel, and lack of or incorrect information (146).

## India's Reactions and Measures to Reduce Stigma

Taking serious consideration of the entrenched stigma associated with the disease in Indian society, the Government of India has been taking active and cognizant measures to curb it. Particularly important is the launch of a caller tune, a public health communication strategy, and appealing to the general public to fight the coronavirus disease, not the diseased. The government has also tried to boost the self-esteem of the doctors, health care professionals, police, and hygiene staff by calling them “corona warriors” and encouraging the general public to pay tribute to the health care professionals. The entire country got together in clapping for the coronavirus warriors from their houses' balconies, they lit up candles outside their houses, and Indian fighter jets showered flowers on the hospitals housing COVID-19 patients. These measures played an important role in reducing stigma and fostering togetherness.

By the end of March, within 15 days of announcing the nationwide lockdown, the Indian Finance Minister, N. Sitharaman, announced INR 1.7 trillion relief package labeled the Pradhan Mantri Garib Kalyan Yojana. It was projected that under the scheme, 800 million Indians would receive 5 kg of wheat and rice for 3 months (in addition to the 5 kg they were already receiving). One kilogram of the preferred pulse was added to this distribution. Furthermore, 60 million farmers registered under the PM-KISAN scheme (who received INR 6,000 per year in three equal installments) were given the first installment upfront for the fiscal year starting April 2020. MNREGA workers' wages were increased from INR 182/— to INR 202/—. The government also provided relief for other marginalized groups, allocating INR 1,000 each for 30 million senior citizens, widows, and disabled Indians and INR 500 per month for 3 months to the 200 million women who were Jan Dhan account holders. Furthermore, women covered under the Ujwala scheme (83 million families) were allocated free LPG cylinders for 3 months. Over 2 crore construction workers received financial support totaling Rs 3,066 crore under the Building and Construction Workers' Fund.

All these actions were aimed at retroactively easing the crisis that was hurled at the Indian working class. At the fore of promising proactive measures to protect these workers is the effort spearheaded by the UP government. They have set up a Migration Commission for the employment of laborers in the state to ensure their social–legal–monetary rights. Any effort toward rehiring workers post lockdown would now require states to seek UP government's permission and follow protective procedures that the commission would outline.

Apart from schemes aimed at helping the working class, the government also delayed the tax filing deadline under “Vivaad Se Vishwas Scheme” from March 31 to June 30, 2020, and expedited the income-tax refunds process, to release all refunds up to INR 0.5 million.

## Summary and Conclusion

The above exposition clearly establishes the deep fissures that underlie the collective, which manifest in times of crises, such as a pandemic. It is rightly said that epidemics reveal who and what is genuinely valued in a society. The power hierarchies come to the fore. This article highlighted stigma associated with being an infected patient, or a close contact of someone infected, along with belonging to a particular race, religion, and social class. It is important to note that stigma reduces health-, help-, and treatment-seeking behavior and needs to be mitigated, apart from the focus on COVID-19 treatment and prevention. Global Health communication plays an important role in the construction of diseases, their social perception, and resulting psychological issues. Thus, all relevant stakeholders, including the government, media and local administrative bodies, as well as hospitals, ought to mitigate stigma through a multipronged approach. Logie and Turan (146) suggest that balancing measures of containment and prevention of the pandemic such as physical distancing and travel restrictions, with appropriate information/public health messages and involvement of communities adversely affected by the pandemic (such as females, LGBTQI, marginalized races, poor), can help reduce the stigma.

Nature has made us all equal. It is us who create divisions in society for our own benefit. Stigmatization serves this purpose. But what it also does is create boundaries at the interpersonal, intergroup, and international levels that are often impossible to undo. There are those who actually commit crimes, and there are also several others who only reap the consequences of being associated with the negative, whether it be in terms of the nature of their work, shared social identity—family, religion—or as simple as being a scapegoat to the injustices that projected their way by the society. What is important to learn from all this is that it reflects a sheer loss of human ability to distinguish between the bad and good and the basic human essence of being kind and helpful toward others. And if this would continue, it is not going to serve any fruitful purpose in the long run for we all are humans first, and the association that we share with our family, religion, profession, socioeconomic status, and many more comes later.

## Author Contributions

DB, TS, SV, and SS conceptualized and collected materials. DB prepared the initial draft. TS and SV reviewed and improved the draft. SS made final revisions. All authors contributed to the article and approved the submitted version.

## Conflict of Interest

The authors declare that the research was conducted in the absence of any commercial or financial relationships that could be construed as a potential conflict of interest.
